# Laryngeal cancer: United Kingdom National Multidisciplinary guidelines

**DOI:** 10.1017/S0022215116000487

**Published:** 2016-05

**Authors:** T M Jones, M De, B Foran, K Harrington, S Mortimore

**Affiliations:** 1 Department of Molecular and Clinical Cancer Medicine, University of Liverpool, Aintree University Hospitals NHS Foundation Trust, Liverpool, UK; 2 Department of ENT–Head and Neck Surgery, Derby Royal Hospitals NHS Foundation Trust, Derby, UK; 3 Sheffield Teaching Hospitals NHS Foundation Trust, Sheffield, UK; 4 The Institute of Cancer Research, Royal Marsden Hospital NHS Trust, London, UK

## Abstract

This is the official guideline endorsed by the specialty associations involved in the care of head and neck cancer patients in the UK. Significantly new data have been published on laryngeal cancer management since the last edition of the guidelines. This paper discusses the evidence base pertaining to the management of laryngeal cancer and provides updated recommendations on management for this group of patients receiving cancer care.

**Recommendations:**

• Radiotherapy (RT) and transoral laser microsurgery (TLM) are accepted treatment options for T1a–T2a glottic carcinoma. (R)

• Open partial surgery may have a role in the management of selected tumours. (R)

• Radiotherapy, TLM and transoral robotic surgery are reasonable treatment options for T1–T2 supraglottic carcinoma. (R)

• Supraglottic laryngectomy may have a role in the management of selected tumours. (R)

• Most patients with T2b–T3 glottic cancers are suitable for non-surgical larynx preservation therapies. (R)

• Concurrent chemoradiotherapy should be regarded as the standard of care for non-surgical management. (R)

• Subject to the availability of appropriate surgical expertise and multi-disciplinary rehabilitation services, TLM or open partial surgical procedures ± post-operative RT, may be also be appropriate in selected cases. (R)

• In the absence of clinical or radiological evidence of nodal disease, elective treatment (RT or surgery ± post-operative RT) is recommended to at least lymph node levels II, III and IV bilaterally. In node positive disease, it is recommended that lymph node levels II–V should be treated on the involved side. If level II nodes are involved, then elective irradiation of ipsilateral level Ib nodes may be considered. (R)

• Most patients with T3 supraglottic cancers are suitable for non-surgical larynx preservation therapies. (R)

• Concurrent chemoradiotherapy should be regarded as the standard of care for non-surgical management. (R)

• Subject to the availability of appropriate surgical expertise and multi-disciplinary rehabilitation services, TLM or open partial surgical procedures ± post-operative RT, may also be appropriate in selected cases. (R)

• In the absence of clinical or radiological evidence of nodal disease, elective treatment (RT or surgery ± post-operative RT) is recommended to at least lymph node levels II, III and IV bilaterally. In node positive disease, lymph node levels II–V should be treated on the involved side. (R)

• As per the PET-Neck clinical trial, patients with N2 or N3 neck disease who undergo treatment with chemoradiotherapy to their laryngeal primary and experience a complete response with a subsequent negative post-treatment positron emission tomography combined with computed tomography (PET–CT) scan do not require an elective neck dissection. In contrast, patients who have a partial response to treatment or have increased uptake on a post-treatment PET–CT scan should have a neck dissection. (R)

• Larynx preservation with concurrent chemoradiotherapy should be considered for T4 tumours, unless there is tumour invasion through cartilage into the soft tissues of the neck, in which case total laryngectomy yields better outcomes. (R)

• In the absence of clinical or radiological evidence of nodal disease, elective treatment (RT or surgery ± post-operative RT) is recommended to bilateral lymph node levels II, III, IV, V and VI. (R)

## Introduction

The aim of any clinician involved in the treatment of laryngeal squamous cell carcinoma should be to cure the disease whilst maintaining maximal laryngeal function. Whilst this seems a simple concept, deciding how best to achieve this aim in any given patient is often difficult and results in well-rehearsed complex discussions within multi-disciplinary team (MDT) meetings throughout the UK on a regular basis. Underpinning this lack of clinical certainty is a lack of level I evidence, particularly with respect to the comparative merits of surgical and non-surgical treatment modalities. Thus, for most laryngeal tumours, perceived treatment equipoise exists. In light of this dearth of good quality comparative data, what treatment any given patient receives is typically related to local MDT dynamics and clinical resources.

Although we are unable to rectify this lack of evidence, in this document we highlight the treatment options available for any given tumour and attempt, based on published evidence, to highlight the relative merits or disadvantages of each approach.

During 2011, 2360 patients were diagnosed with laryngeal carcinoma in the UK. Of these, 1506, 108, 245 and 73 were diagnosed in England, Wales, Scotland and Northern Ireland, respectively. Accordingly, European Age Standardised Rates per 100 000 for England, Wales, Scotland and Northern Ireland are 2.7, 3.0, 4.2 and 4.3, respectively; highlighting the fact that larynx cancer is more common in Wales, Scotland and Northern Ireland. For the UK as a whole, 1932 (82 per cent) cases occurred in men and 428 (18 per cent) in women (M:F; 4.5:1). Larynx cancer accounts for 1 per cent of all cancers in men and 0.3 per cent of all cancers in women. However, this amounts to a 22 per cent reduction of cases diagnosed in men when comparing 1992–1994 with 2009–2011. A comparable reduction (19 per cent) has occurred in women over this timeframe. (http://info.cancerresearchuk.org/cancerstats/types/larynx) in keeping with the geographical variation in incidence, larynx cancer is more commonly diagnosed in patients of lower socio-economic groups.^1^

It is well documented that alcohol and tobacco, separately and synergistically are the main causes of larynx cancer. However, in contrast to oropharynx cancer, it appears that human papilloma virus infection is not a major cause.^2^

Larynx cancer is rare in patients younger than 40 years of age, with incidence increasing with age, rising to a peak in the eighth decade. Three-quarters of all diagnoses occur in patients older than 60 years. (http://info.cancerresearchuk.org/cancerstats/types/larynx)

In 2012, 618 men (79 per cent) and 166 women (21 per cent) died of larynx cancer (M:F; 3.7:1). This constitutes a marked decrease – 25 and 16 per cent, respectively – in age-standardised mortality for men and women over the last decade. (http://info.cancerresearchuk.org/cancerstats/types/larynx)

However, Rachet *et al*.^1^ previously demonstrated a startling differential mortality rate between socio-economic groups, with patients from lower socio-economic groups suffering higher death rates from larynx cancer than those from higher socio-economic groups.

## Clinical presentation

The clinical presentation of laryngeal cancer is highly variable and depends on the site and size of the primary tumour. Tumours of the glottis, for example, typically present at an early stage as they manifest as hoarseness. In comparison, tumours of the supraglottis are likely to present later with symptoms of pain, hoarseness or swallowing difficulty. However, it is not uncommon for patients presenting with laryngeal cancer to delay seeking medical advice on developing ‘early’ symptoms, only to present at a much later stage with symptoms of pain, swallowing difficulty, a palpable neck mass or even, in extreme cases, with airway compromise.

## Assessment and staging

As with all head and neck cancers, diagnosis of laryngeal cancer relies initially on good history taking and clinical examination in the clinic. Laryngeal cancers are, in most cases, obvious following inspection of the larynx with a fibreoptic laryngoscope in the outpatient department. Initial assessment of the tumour stage relies on imaging. Whilst exact protocols vary according to local imaging preferences, it is typical for patients suspected of having laryngeal cancer to undergo either magnetic resonance imaging or computed tomography (CT) of the head and neck and CT scan of the thorax and upper abdomen. The exception to this is in patients presenting with the early stage, T1 lesions of the glottis without anterior commissure involvement, where imaging is unhelpful. Definitive diagnosis is achieved by histological examination of a tissue biopsy, obtained usually at the time of a general anaesthetic endoscopic examination of the larynx, pharynx and upper oesophagus. The examination under anaesthesia is extremely important for staging and should routinely involve inspection with rigid (plane 0° and angled 30° and/or 70°) fibreoptic endoscopes. The aggregate information provided by the imaging and the endoscopic examination facilitates the staging of the tumour according to the tumour–node–metastasis (TNM) system outlined below (Table I). It is by recourse to the TNM stage of the tumour, in addition to the general fitness of the patient, that treatment decisions are ultimately made.

**Table I:** TNM Staging system for laryngeal cancer

Supraglottis	
T1	Tumour limited to one subsite of supraglottis with normal vocal fold mobility
T2	Tumour invades mucosa of more than one adjacent subsite of supraglottis or glottis or region outside the supraglottis (e.g. mucosa of base of tongue, vallecula, medial wall of piriform sinus) without fixation of the larynx
T3	Tumour limited to larynx with vocal fold fixation and/or invades any of the following: post-cricoid area, pre-epiglottic tissues, paraglottic space, and/or with minor thyroid cartilage erosion (e.g. inner cortex)
T4a	Tumour invades through the thyroid cartilage and/or invades tissues beyond the larynx, e.g. trachea, soft tissues of neck, including deep/extrinsic muscle of tongue (e.g. genioglossus, hyoglossus, palatoglossus and styloglossus), strap muscles, thyroid and oesophagus
T4b	Tumour invades pre-vertebral space, mediastinal structures or encases carotid artery
Glottis	
T1	Tumour limited to vocal fold(s) (may involve anterior or posterior commissure) with normal mobility T1a. Tumour limited to one vocal fold T1b. Tumour involves both vocal folds
T2	T2a. Tumour extends to supraglottis and/or subglottis with normal vocal fold mobility T2b. Tumour extends to supraglottis and/or subglottis with impaired vocal fold mobility
T3	Tumour limited to larynx with vocal fold fixation and/or invades paraglottic space, and/or with minor thyroid cartilage erosion (e.g. inner cortex)
T4a	Tumour invades through the thyroid cartilage or invades tissues beyond the larynx, e.g. trachea, soft tissues of neck, including deep/extrinsic muscle of tongue (genioglossus, hyoglossus, palatoglossus and styloglossus), strap muscles, thyroid and oesophagus
T4b	Tumour invades prevertebral space, mediastinal structures or encases carotid artery
Subglottis	
T1	Tumour limited to subglottis
T2	Tumour extends to vocal fold(s) with normal or impaired mobility
T3	Tumour limited to larynx with vocal fold fixation
T4a	Tumour invades through cricoid or thyroid cartilage and/or invades tissues beyond the larynx, e.g., trachea, soft tissues of neck including deep/extrinsic muscle of tongue (genioglossus, hyoglossus, palatoglossus and styloglossus), strap muscles, thyroid and oesophagus
T4b	Tumour invades prevertebral space, mediastinal structures or encases carotid artery

## Management

### Early (T1–T2a) glottic carcinoma

Early laryngeal cancer (T1–T2a N0 M0) is characterised by low tumour volume and a low incidence of metastatic neck disease. Consequently, the chances of cure are extremely good whichever of the main treatment options – radiotherapy (RT), transoral laser microsurgery (TLM) or open partial laryngeal surgery – is employed. A systemic review^3^ has confirmed there is insufficient evidence to determine which of these three treatment options is most effective for the treatment of early glottic carcinoma.

Radiotherapy with surgery in reserve or TLM are the two most commonly used treatment modalities in the UK. Whilst survival outcomes and local control rates are similar,^4^ they have not been compared in randomised trials. Individual treatment selection depends on patient and tumour factors (e.g. indistinct tumours diffusely infiltrating the vocal fold mucosa and larger volume tumours involving the anterior commissure may be more suitable for RT than transoral laser surgery) and local expertise. Single-modality treatment is sufficient and combining surgery with RT should be avoided as functional outcomes (and perhaps survival in the context of incompletely resected tumour) may be compromised by combined-modality therapy. Radiotherapy is delivered using megavoltage photons from a linear accelerator (typical energies 4–6 MV); hypofractionated RT schedules, using a fraction size greater than 2 Gray (Gy), results in equivalent outcomes to longer schedules, without increased toxicity. Typical schedules include 50–52 Gy in 16 fractions and 53–55 Gy in 20 fractions over three to four weeks.^5^ Elective treatment of the neck is not recommended because of the very low risk of occult nodal disease. Radiotherapy results in significant acute toxicity, including thick, sticky secretions, hoarse voice, odynophagia and skin reactions. Most of these effects resolve four to six weeks after the completion of treatment and significant late effects are rare. Should tumour recurrence occur, partial laryngeal surgery provides a salvage option in appropriate clinical settings, resulting in good oncological and functional outcomes. However, these techniques are rarely offered in the UK and, therefore, total laryngectomy is most commonly performed.

Transoral laser microsurgery is usually undertaken using a CO_2_ laser as a day case procedure and has minimal acute morbidity. Whilst there is equipoise with respect to voice outcome between RT and TLM for smaller tumours, long-term quality of voice for T2 glottic cancers is generally accepted to be better after RT than after TLM. Voice outcome following TLM is dependent on the extent of the resection and/or whether the resection includes the anterior commissure.^4^ Certain patient factors, may preclude TLM, such as restriction of neck movement and difficult access. In these patients, hypofractionated RT is the preferred option.

Contrary to the practice in other countries, in the UK, partial open surgical procedures are used less commonly for the treatment of early *de novo* glottic carcinoma. However, they provide an option for the treatment of *de novo* tumours which are not accessible to TLM and for recurrent tumours after TLM or RT. Meta-analysis data show similar rates of local control and survival after partial laryngectomy (comparable with TLM and RT) with larynx preservation rates of 98.3 per cent for *de novo* tumours and 84.6 per cent for radio-recurrent tumours.^6^^,^^7^ Open surgical procedures include laryngofissure cordectomy, vertical partial laryngectomy (VPL) ± reconstruction, frontolateral vertical partial laryngectomy, supraglottic laryngectomy, supracricoid partial laryngectomy plus cricohyoidoepiglottopexy or cricohyoidopexy reconstruction (SCPL–CHEP or CHP) and extended supraglottic laryngectomy.

Overall, for T1a glottic tumours the local control is similar between RT and TLM (five-year local control rate 90–93 per cent). In the case of T1b disease, the local control rate is lower (85–89 per cent).

Similarly, the local control and overall survival rates for T2a glottic cancers are comparable when treated with TLM, partial laryngeal resection or RT.

**Recommendations**

• Radiotherapy and transoral laser microsurgery are accepted treatment options for T1a–T2a glottic carcinoma (R)
• Open partial surgery may have a role in the management of selected tumours (R)

### T1–T2 supraglottic cancers

Radiotherapy, TLM and transoral robotic surgery (TORS) are valid treatment options for all patients with T1–T2 supraglottic cancers. As with glottic carcinomas, open partial surgical procedures (supraglottic laryngectomy) are used less commonly in the UK but open supraglottic laryngectomy may have a role in selected cases in units with appropriate surgical expertise and multi-disciplinary support services. Survival outcomes appear to be similar with RT and surgery although, once again, there are no randomised comparative data. Whilst long-term functional (voice and swallowing) outcomes appear similar, early swallowing function is usually poorer after surgery: swallowing rehabilitation may be prolonged and in a small proportion of patients, adequate swallowing function may never be achieved. Consequently, patient selection, based on tumour burden and performance status, is imperative. Again, every effort should be made to avoid combining surgery with RT because functional outcomes may be compromised by combined-modality therapy.

The supraglottis has a rich lymphatic supply and, as a consequence, the risk of nodal disease is significantly higher for T1–T2 supraglottic cancers than for T1–T2 glottic cancers. Thus, even in the absence of clinical or radiological evidence of nodal involvement, elective treatment of at least bilateral lymph node levels II and III – either with RT or selective neck dissection – is recommended.

Whilst RT or surgery alone, is sufficient for the treatment of node negative T1–T2 supraglottic cancers, concurrent platinum-based chemoradiotherapy or surgery followed by post-operative RT is recommended for node positive supraglottic carcinoma (T1–T2 N1+, stage III–IV) in patients whose performance status is sufficient to tolerate this treatment. The role of induction chemotherapy prior to chemoradiotherapy or surgery remains unclear but may be appropriate for patients presenting with advanced nodal disease (e.g. N2c/N3), particularly if this is rapidly progressive and/or symptomatic.

*All treatment options appear to effect similar locoregional control and survival rates*: For T1 disease, five-year local control rates following treatment with RT, TLM, TORS or open supraglottic laryngectomy range from 77 to 100 per cent. For T2 tumours, the five-year local control rates range from 80 to 97 per cent for TLM or open supraglottic laryngectomy and from 62 to 83 per cent for primary RT.^8^

**Recommendations**

• Radiotherapy, transoral laser microsurgery and transoral robotic surgery are reasonable treatment options for T1–T2 supraglottic carcinoma (R)
• Supraglottic laryngectomy may have a role in the management of selected tumours (R)

### T2b–T3 glottic tumours

Most patients with T2b–T3 glottic cancers are suitable for radiation-based larynx preservation therapy. However, subject to the availability of appropriate surgical expertise and multi-disciplinary rehabilitation services, TLM or open partial surgical procedures ± post-operative RT, may also be appropriate in selected cases. Open partial surgical procedures which might be considered include VPL ± reconstruction, frontolateral VPL, supraglottic laryngectomy, SCPL–CHEP or CHP and extended supraglottic laryngectomy. In the absence of clinical or radiological evidence of nodal disease, elective treatment (RT or surgery ± post-operative RT) is recommended to at least lymph node levels II, III and IV bilaterally, because of the risk of occult nodal metastasis. Intensity-modulated radiotherapy (IMRT) allows a convenient solution to elective nodal treatment, enabling differential doses of RT to be given to different nodal groups simultaneously, depending on the presence or absence of macroscopic disease and the risk of subclinical disease.

In node positive disease, it is recommended that lymph node levels II-V should be treated on the involved side. If level II nodes are involved, then elective irradiation of ipsilateral level Ib nodes may be considered.

The potential of RT and chemotherapy for larynx preservation was established by the landmark Veterans Affairs Laryngeal Cancer Study Group (VALCSG) study^9^ in which induction chemotherapy and RT (IC + RT) yielded similar overall survival (68 per cent at two years) to laryngectomy followed by adjuvant RT for stage III–IV laryngeal cancer with high rates of larynx preservation (64 per cent at two years). Rates of salvage laryngectomy were significantly lower for T3 *vs* T4 disease (29 per cent *vs* 56 per cent, *p* = 0.001). Subsequently, the RTOG (Radiation Therapy Oncology Group) 91-11 trial^10^ demonstrated that concurrent chemoradiotherapy was superior to IC + RT and RT alone in terms of laryngeal preservation (88 *vs* 75 *vs* 70 per cent, respectively, at three years), although overall survival in each treatment arm was similar. Of note, 10-year follow-up data have confirmed the superiority of concurrent chemoradiotherapy, but a significant increase in non-cancer deaths in the group treated with chemoradiotherapy was reported.^11^ The use of concurrent chemoradiotherapy for locally advanced head and neck cancers, including laryngeal cancers, is also supported by meta-analysis data.^12^ Standard concurrent chemotherapy regimens include cisplatin (100 mg/m^2^) on days 1, 22 and 43 of RT and carboplatin/5-FU on weeks 1 and 5 of RT.

Concurrent chemoradiotherapy is, however, associated with a significant increase in acute and late toxicity compared with RT alone. The long-term side effects of chemoradiotherapy are well documented: 43 per cent of patients develop severe (grade III/IV) late toxicity, including a reduction in speech and swallowing function which can lead to life-long dependence on a feeding tube (13 per cent of patients two years after treatment) and have a profound effect on quality of life (QoL).^13^ (Although these late severe toxicities are likely to affect fewer patients when contemporary RT delivery schedules are used.) Older age, advanced T stage, larynx/hypopharynx primary site and neck dissection after chemoradiotherapy all increase the risk of severe late toxicity after chemoradiotherapy and the additional benefit of chemotherapy must be balanced against the risks for individual patients. The benefit of chemotherapy decreases with age and is non-significant above 70 years of age. Thus, its use may be less appropriate in older patients. Other systemic therapies that may be given concurrently with RT include cetuximab, a monoclonal antibody which competitively inhibits the cell-surface epidermal growth factor receptor. Cetuximab has been shown to improve locoregional control (three-year LRC 47 *vs* 34 per cent, *p* < 0.01) and overall survival (by 10 per cent – three-year OS 55 *vs* 45 per cent) over RT alone in a study of patients with locally advanced (stage III/IV) head and neck cancer (27 per cent of whom had laryngeal cancer). The benefit was maintained on longer follow-up (five-year OS 46 *vs* 36 per cent).^14^ Toxicities of cetuximab include an acneiform rash and hypersensitivity reactions but it does not increase the rate of severe radiation-related mucositis. It is an alternative to concurrent chemoradiotherapy for patients with laryngeal cancer who cannot receive concurrent chemoradiotherapy, as per the guidelines published in 2008 by the National Institute for Health and Care Excellence (http://www.nice.org.uk/guidance/ta145).

Induction chemotherapy with cisplatin and 5-FU (PF) prior to RT may also improve survival,^15^ but the benefit of induction chemotherapy prior to standard concurrent chemoradiotherapy schedules is currently unproven. If induction chemotherapy is used, taxane (docetaxel or paclitaxel) in combination with cisplatin and 5-FU has been shown to be superior to PF doublet chemotherapy in a meta-analysis of five randomised trials.^16^

Radiotherapy may be used as a single modality where comorbidity precludes the use of concurrent chemotherapy, cetuximab or surgery. Conventional RT alone may be suboptimal for the treatment of advanced laryngeal cancer. Altered fractionation regimens (including acceleration and hyperfractionation) improve locoregional control and overall survival compared with standard fractionated RT for head and neck cancer patients who elect or are selected to receive RT alone (albeit at the cost of higher mucosal toxicity).^17^ However, altered fractionation regimens do not appear to improve outcome compared with or when combined with concurrent chemoradiotherapy which should be regarded as the ‘standard of care’ for the non-surgical management of advanced laryngeal cancer. Accelerated fractionation with hypoxia modification using either nimorazole or carbogen/nicotinamide shows promising results and requires further study. To that end, the UK clinical trial NIMRAD (a randomised placebo-controlled trial of synchronous NIMorazole *vs* RADiotherapy alone in patients with locally advanced head and neck squamous cell carcinoma not suitable for synchronous chemotherapy or Cetuximab) (NCT01950689) is currently recruiting in several UK centres.

It is important to note that, despite the laryngeal preservation and survival rates conferred by non-surgical strategies, there is a dearth of robust data relating to laryngeal function after chemoradiotherapy. By comparison with non-surgical treatments, any larynx-preserving surgical procedure – TLM or partial open procedure – undertaken for T2b/T3 carcinoma of the larynx will result in dysphonia and prolonged swallowing rehabilitation. Although most patients appear to achieve satisfactory swallowing function eventually, a small percentage of patients will require a total laryngectomy for functional reasons.

Whilst TLM or partial open surgical procedures may be considered as an alternative to non-surgical treatment for selected cases in appropriate centres, laryngectomy may be preferred for patients with significant pre-existing laryngeal destruction by tumour and/or a pre-treatment tracheostomy; however, reports of whether a pre-treatment tracheostomy negatively affects outcome after RT are conflicting and concurrent chemoradiotherapy remains an option for these patients (25 per cent of patients in the VALCSG study^9^ had a baseline tracheostomy and they were not excluded from RTOG 91-11). Vocal cord fixation is not a contraindication to larynx preservation (for either surgical or non-surgical modalities), although it is likely that these patients will have a poorer functional and oncological outcome than patients with mobile vocal folds.

In the absence of clinical or radiological evidence of nodal disease, elective treatment (RT or surgery ± post-operative RT) is recommended to at least lymph node levels II, III and IV bilaterally.

**Recommendations**

• Most patients with T2b–T3 glottic cancers are suitable for non-surgical larynx preservation therapies (R)
• Concurrent chemoradiotherapy should be regarded as the standard of care for non-surgical management (R)
• Subject to the availability of appropriate surgical expertise and multi-disciplinary rehabilitation services, TLM or open partial surgical procedures ± post-operative RT, may also be appropriate in selected cases (R)
• In the absence of clinical or radiological evidence of nodal disease, elective treatment (RT or surgery ± post-operative RT) is recommended to at least lymph node levels II, III and IV bilaterally. In node positive disease, it is recommended that lymph node levels II–V should be treated on the involved side. If level II nodes are involved, then elective irradiation of ipsilateral level Ib nodes may be considered (R)

### T3 supraglottic carcinoma

The principles of organ preservation for T3 supraglottic cancers are the same as for glottic cancers. Tumour size, pre-treatment laryngeal function and performance status should direct the management of individual patients. Rates of salvage laryngectomy after surgical and non-surgical treatment of supraglottic cancers are lower than for glottic cancers. Vocal cord function is usually well preserved following TLM or supraglottic laryngectomy; however, rehabilitation of swallowing function following supraglottic surgery may be prolonged and, whilst most patients achieve satisfactory swallowing function, this cannot be guaranteed.

T3 supraglottic cancers have a significantly higher risk of nodal disease (occult and clinical) than glottic tumours and this must be taken into account when considering how to manage the neck. In the absence of clinical or radiological evidence of nodal disease, elective treatment – RT and/or selective neck dissection – is recommended to at least lymph node levels II, III, IV bilaterally.

There is general agreement that chemoradiotherapy is sufficient to treat early nodal disease (N1, single lymph node <3 cm) in patients with glottic cancers. Since publication of the last edition, management of N2 (multiple lymph nodes and/or >3–6 cm) or N3 (>6 cm) nodal disease has been informed by the PET-Neck clinical trial.^18^ The data confirm that positron emission tomography combined with computed tomography (PET–CT) surveillance of the neck in chemoradiotherapy complete responders, obviates the need for an elective neck dissection in patients with a negative PET–CT scan result.

**Recommendations**

• Most patients with T3 supraglottic cancers are suitable for non-surgical larynx preservation therapies (R)
• Concurrent chemoradiotherapy should be regarded as the standard of care for non-surgical management (R)
• Subject to the availability of appropriate surgical expertise and multi-disciplinary rehabilitation services, TLM or open partial surgical procedures ± post-operative RT, may also be appropriate in selected cases (R)
• In the absence of clinical or radiological evidence of nodal disease, elective treatment (RT or surgery ± post-operative RT) is recommended to at least lymph node levels II, III and IV bilaterally. In node positive disease, lymph node levels II–V should be treated on the involved side (R)
• As per the PET-Neck clinical trial, patients with N2 or N3 neck disease who undergo treatment with chemoradiotherapy to their laryngeal primary and experience a complete response with a subsequent negative post-treatment PET–CT scan do not require planned neck dissection. In contrast, patients who have a partial response to treatment or have increased uptake on a post-treatment PET–CT scan should have a neck dissection (R)

### T4 laryngeal carcinoma

Larynx preservation with chemoradiotherapy should be considered for T4 tumours, unless there is tumour invasion through cartilage into the soft tissues of the neck, in which total laryngectomy followed by adjuvant treatment yields better outcomes. The VALCSG study^9^ showed reduced tumour response to chemotherapy and higher rates of salvage laryngectomy for T4 tumours (56 per cent for T4 *vs* 29 per cent for T3 tumours, *p* = 0.001). Nevertheless, larynx preservation can be achieved in a significant proportion of patients with T4 disease, without detriment to survival when salvage laryngectomy is incorporated. However, once again, few data are available correlating laryngeal preservation with function and QoL. Good patient selection is of paramount importance. Patients with large-volume T4 tumours – defined as extension of tumour through thyroid cartilage or tumour extension greater than 1 cm into the base of tongue – were excluded from RTOG 91-11^10^ as they are poor candidates for organ preservation. Patients with significant pre-existing laryngeal destruction by tumour and/or a pre-treatment tracheostomy may also be better suited to a total laryngectomy. Total laryngectomy may confer a better QoL than a preserved, but poorly functioning, larynx.

Patients with large-volume T4 tumours who are unsuitable for surgery because of inoperable (T4b) disease have been treated with combined-modality organ preservation therapy with significant rates of disease control (71 per cent at four years) and overall survival (56 per cent at four years) in retrospective studies. Induction chemotherapy may be used to treat large volume, symptomatic disease prior to commencement of concurrent chemoradiotherapy.

Lymph node levels II–V bilaterally should be treated, irrespective of the pre-treatment clinical nodal status. As per the findings of the PET-Neck trial^18^ (see above), a planned neck dissection is not necessary in patients who experience a complete response to chemoradiotherapy and have a post-treatment negative PET–CT scan. Improved systemic therapies and RT dose intensification using IMRT may improve outcomes for this patient group in future.

**Recommendations**

• Larynx preservation with concurrent chemoradiotherapy should be considered for T4 tumours, unless there is tumour invasion through cartilage into the soft tissues of the neck, in which case total laryngectomy yields better outcomes (R)
• In the absence of clinical or radiological evidence of nodal disease, elective treatment (RT or surgery ± post-operative RT) is recommended to bilateral lymph node levels II, III, IV, V and VI (R)

### Post-operative RT /chemoradiotherapy

Radiotherapy delivered post-operatively to the primary site and/or neck in patients at high risk of locoregional recurrence can improve locoregional control and survival. Post-operative RT is recommended for pT4 laryngeal cancers of any nodal stage, pT1/T2/T3 tumours with N2–N3 nodal stage and for all patients with close or positive resection margins and/or extracapsular spread; other unfavourable pathological factors, including peri-neurial and vascular invasion, are relative indications for post-operative RT. Administration of concurrent cisplatin chemotherapy with post-operative RT improves locoregional control and disease-free survival compared with post-operative RT alone for locally advanced tumours,^19^^,^^20^ albeit at the expense of increased mucosal and haematological toxicity and possibly increased deaths. This approach improves overall survival in selected patients, particularly with extracapsular spread and/or positive margins, and should be used selectively for patients at highest risk of relapse.

### Key points

• Approximately 2400 patients are diagnosed with laryngeal squamous cell carcinoma and ~800 patients die of the disease per annum in the UK
• Early stage tumours of the glottis present with hoarseness, whilst tumours of the supraglottis and more advanced glottic tumours may present with pain, odynophagia and/or dysphagia, a neck lump or even airway compromise
• Meticulous endoscopic inspection of the tumour under general anaesthetic and imaging of the head, neck and thorax is needed for staging
• Radiotherapy and transoral laser microsurgery are reasonable treatment options for T1a–T2a glottic and T1–T2 supraglottic carcinomas
• Most patients with T2b–T3 glottic and T3 supraglottic cancers are suitable for non-surgical larynx preservation therapies. Transoral laser microsurgery or open partial surgical procedures ± post-operative radiotherapy may be also be appropriate in selected cases
• Concurrent chemoradiotherapy should be regarded as standard of care for the non-surgical management of stage III/IV laryngeal cancer
• Patients with N2 or N3 neck disease who experience a complete response with a subsequent negative post-treatment PET–CT scan do not require planned neck dissection
• Post-operative (chemo)radiotherapy is recommended in the presence of advanced disease or adverse histological features.

## References

[1] Rachet B, Quinn MJ, Cooper N, Coleman MP. Survival from cancer of the larynx in England and Wales up to 2001. Br J Cancer 2008;99(Suppl 1):S35–7 18813254 10.1038/sj.bjc.6604581 PMC2557544

[2] Upile NS, Shaw RJ, Jones TM, Goodyear P, Liloglou T, Risk JM Squamous cell carcinoma of the head and neck outside the oropharynx is rarely human papillomavirus related. Laryngoscope 2014;124:2739–44 25042603 10.1002/lary.24828

[3] Dey P, Arnold D, Wight R, MacKenzie K, Kelly C, Wilson J. Radiotherapy *versus* open surgery *versus* endolaryngeal surgery (with or without laser) for early laryngeal squamous cell cancer. Cochrane Database Syst Rev 2002;CD002027 12076435 10.1002/14651858.CD002027

[4] Steiner W, Ambrosch P, Rodel RM, Kron M. Impact of anterior commissure involvement on local control of early glottic carcinoma treated by laser microresection. Laryngoscope 2004;114:1485–91 15280731 10.1097/00005537-200408000-00031

[5] Gowda RV, Henk JM, Mais KL, Sykes AJ, Swindell R, Slevin NJ. Three weeks radiotherapy for T1 glottic cancer: the Christie and Royal Marsden Hospital Experience. Radiother Oncol 2003;68:105–11 12972304 10.1016/s0167-8140(03)00059-8

[6] Thomas LD, Basavaiah M, Mehanna N, Jones H, Paleri V. Open Conservation partial Laryngectomy for laryngeal cancer: a Systematic review of English language literature. Cancer Treat Rev 2012;38:203–11. 21764220 10.1016/j.ctrv.2011.05.010

[7] Paleri V, Thomas L, Basavaiah N, Drinnan M, Mehanna H, Jones T. Oncologic outcomes of open conservation laryngectomy for radiorecurrent laryngeal carcinoma: a systematic review and meta-analysis of English-language literature. Cancer 2011;117:2668–76 21287526 10.1002/cncr.25831

[8] Ambrosch P. The role of laser microsurgery in the treatment of laryngeal cancer. Curr Opin Otolaryngol Head Neck Surg 2007;15:82–8 17413407 10.1097/MOO.0b013e3280147336

[9] The Department of Veterans Affairs Laryngeal Cancer Study Group. Induction chemotherapy plus radiation compared with surgery plus radiation in patients with advanced laryngeal cancer. N Engl J Med 1991;324:1685–90 2034244 10.1056/NEJM199106133242402

[10] Forastiere AA, Goepfert H, Maor M, Pajak TF, Weber R, Morrison W Concurrent chemotherapy and radiotherapy for organ preservation in advanced laryngeal cancer. N Engl J Med 2003;349:2091–8 14645636 10.1056/NEJMoa031317

[11] Forastiere AA, Zhang Q, Weber RS, Maor MH, Goepfert H, Pajak TF Long-term results of RTOG 91-11: a comparison of three nonsurgical treatment strategies to preserve the larynx in patients with locally advanced larynx cancer. J Clin Oncol 2013;31:845–52 23182993 10.1200/JCO.2012.43.6097 PMC3577950

[12] Pignon JP, le Maitre A, Maillard E, Bourhis J. Meta-analysis of chemotherapy in head and neck cancer (MACH-NC): an update on 93 randomised trials and 17,346 patients. Radiother Oncol 2009;92:4–14 19446902 10.1016/j.radonc.2009.04.014

[13] Machtay M, Moughan J, Trotti A, Garden AS, Weber RS, Cooper JS Factors associated with severe late toxicity after concurrent chemoradiation for locally advanced head and neck cancer: an RTOG analysis. J Clin Oncol 2008;26:3582–9 18559875 10.1200/JCO.2007.14.8841 PMC4911537

[14] Bonner JA, Harari PM, Giralt J, Cohen RB, Jones CU, Sur RK Radiotherapy plus cetuximab for locoregionally advanced head and neck cancer: 5-year survival data from a phase 3 randomised trial, and relation between cetuximab-induced rash and survival. Lancet Oncol 2010;11:21–8 19897418 10.1016/S1470-2045(09)70311-0

[15] Monnerat C, Faivre S, Temam S, Bourhis J, Raymond E. End points for new agents in induction chemotherapy for locally advanced head and neck cancers. Ann Oncol 2002;13:995–1006 12176777 10.1093/annonc/mdf172

[16] Blanchard P, Bourhis J, Lacas B, Posner MR, Vermorken JB, Hernandez JJ Taxane-cisplatin-fluorouracil as induction chemotherapy in locally advanced head and neck cancers: an individual patient data meta-analysis of the meta-analysis of chemotherapy in head and neck cancer group. J Clin Oncol 2013;31:2854–60 23835714 10.1200/JCO.2012.47.7802

[17] Bourhis J, Overgaard J, Audry H, Ang KK, Saunders M, Bernier J Hyperfractionated or accelerated radiotherapy in head and neck cancer: a meta-analysis. Lancet 2006;368:843–54 16950362 10.1016/S0140-6736(06)69121-6

[18] Mehanna H, Wong WL, McConkey CC, Rahman J, Robinson M, Hartley A PETCT surveillance versus neck dissection in advanced head and neck cancer. N Engl J Med 2016;374:1444–54. 27007578 10.1056/NEJMoa1514493

[19] Bernier J, Domenge C, Ozsahin M, Matuszewska K, Lefèbvre JL, Greiner RH Postoperative irradiation with or without concomitant chemotherapy for locally advanced head and neck cancer. N Engl J Med 2004;350:1945–52 15128894 10.1056/NEJMoa032641

[20] Cooper JS, Pajak TF, Forastiere AA, Jacobs J, Campbell BH, Saxman SB Postoperative concurrent radiotherapy and chemotherapy for high-risk squamous-cell carcinoma of the head and neck. N Engl J Med 2004;350:1937–44 15128893 10.1056/NEJMoa032646

